# Supplementary material for: PDL2+ CD11b+ dermal dendritic cells capture topical antigen through hair follicles to prime LAP+ Tregs
Source: Nat Commun. 2018 Dec 7;9:5238. doi: 10.1038/s41467-018-07716-7 (PMC6286332; doi:10.1038/s41467-018-07716-7)
Supplement: Supplementary file 1 — Supplementary Information [file 41467_2018_7716_MOESM1_ESM.pdf]

Supplementary Information

"PDL2$^+$ CD11b$^+$ dermal dendritic cells capture topical antigen through hair follicles to prime LAP$^+$ Tregs"

Tordesillas, et al

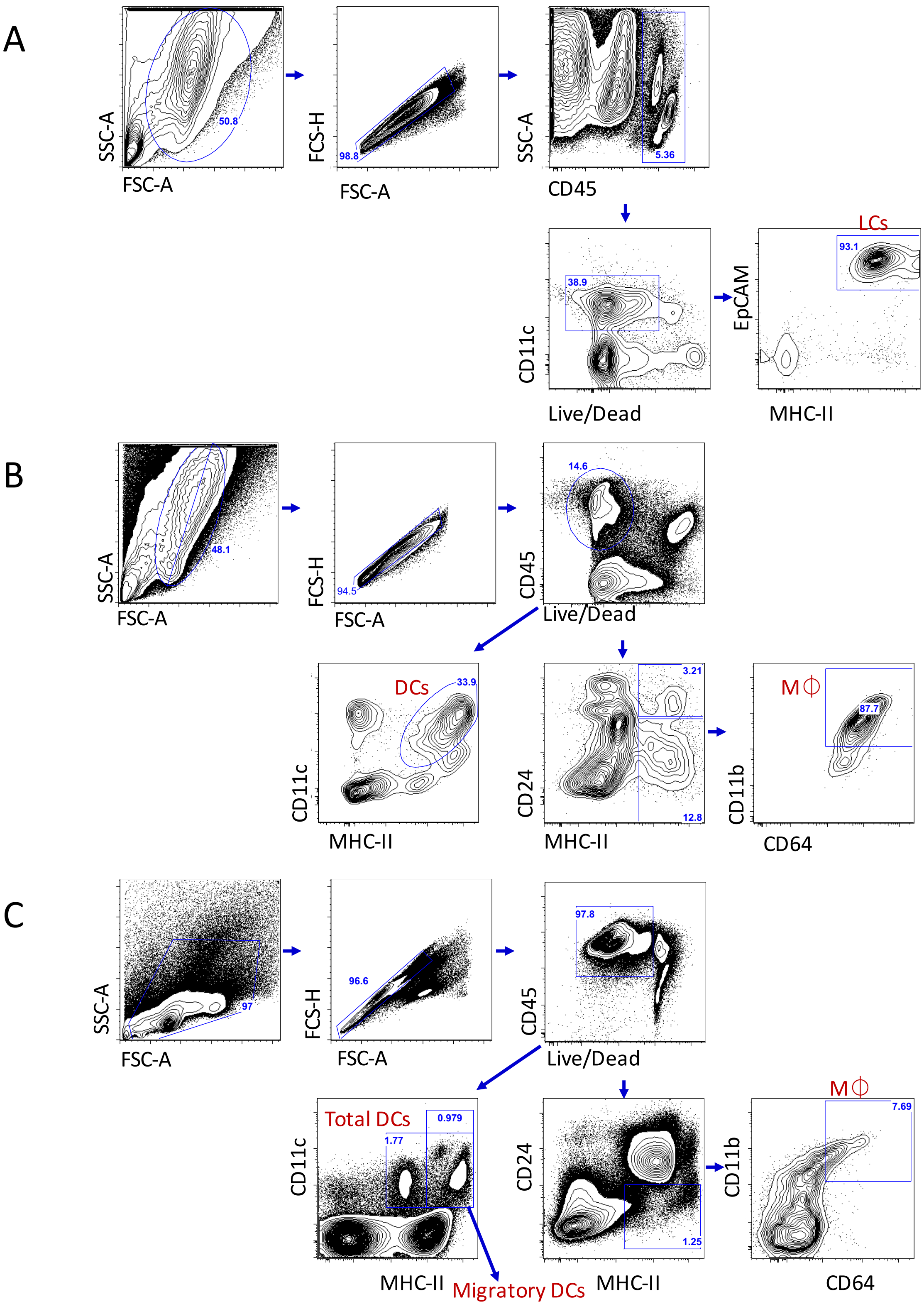

Supplementary Figure 1: Gating strategy of LCs, dermal DCs and macrophages. *Representative plots showing gating strategy of LCs in epidermis (A, used in Figures 1A,4A,4C,6C,8B), DCs and macrophages in dermis (B, used in Figure 1B), and total and migratory DCs and macrophages in lymph node (C, used in Figure 2D).*

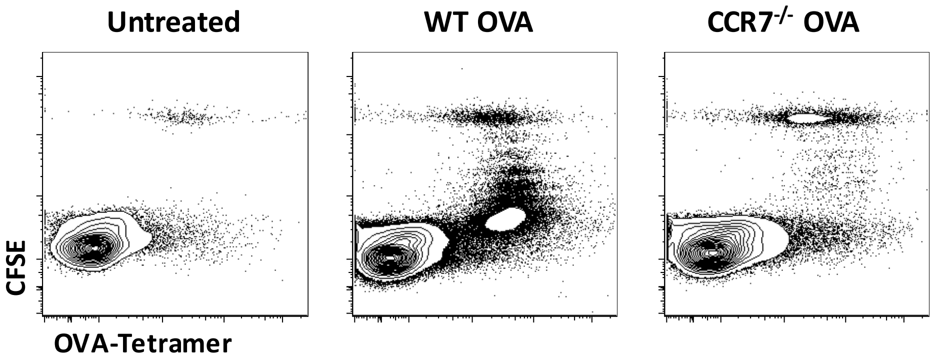

Supplementary Figure 2. Topical antigen presentation is decreased in the absence of migratory DCs. Representative plots of proliferation of OT-II cells (OVA-tetramer$^+$ cells) CD4$^+$ T cells recovered from skin-draining lymph nodes from wild-type C57BL/6 or CCR7$^{-/-}$ mice treated with an OVA patch for 48h. Cells were recovered three days after patch application. Representative of 3 mice per group.

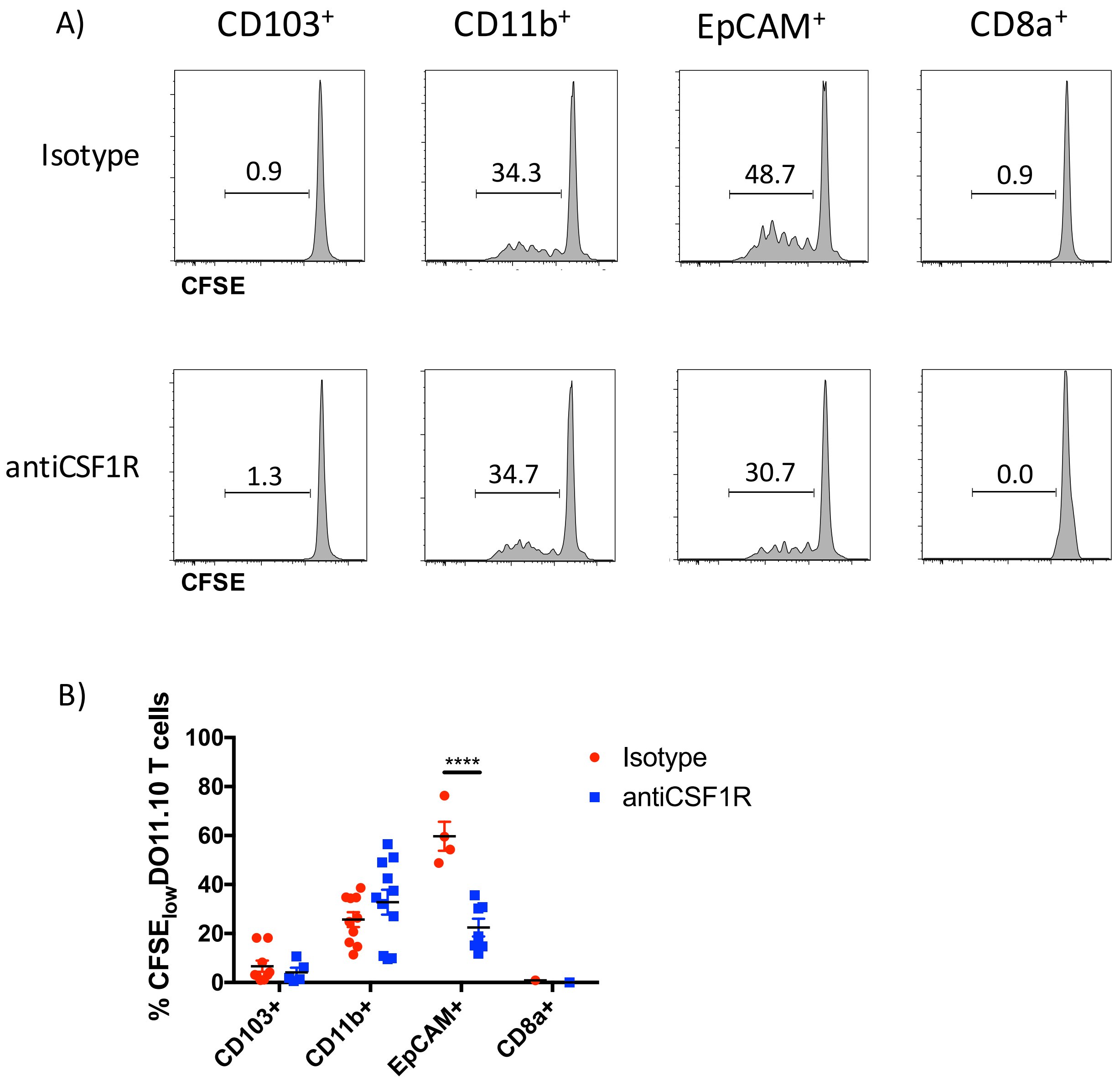

Supplementary Figure 3. Topical antigen presentation in LCs-depleted mice. (A) Representative plots of proliferation of DO11.10 CD4+ T cells after co-culture with sorted DC subsets from skin-draining lymph nodes from mice treated with anti-CSF1R antibody (blue squares) or isotype control (red circles). (B) Percentage of $CFSE_{low}$DO11.10 CD4+ T cells in the co-cultures, from mice treated with OVA patch for 48h. Data are mean ± s.e.m. of 4 or more replicates for populations CD103+, EpCAM+ and CD11b+. 1 replicates is shown for CD8a+. ****$p < 0.0001$, Two-way Anova, Sidak's multiple comparisons test.

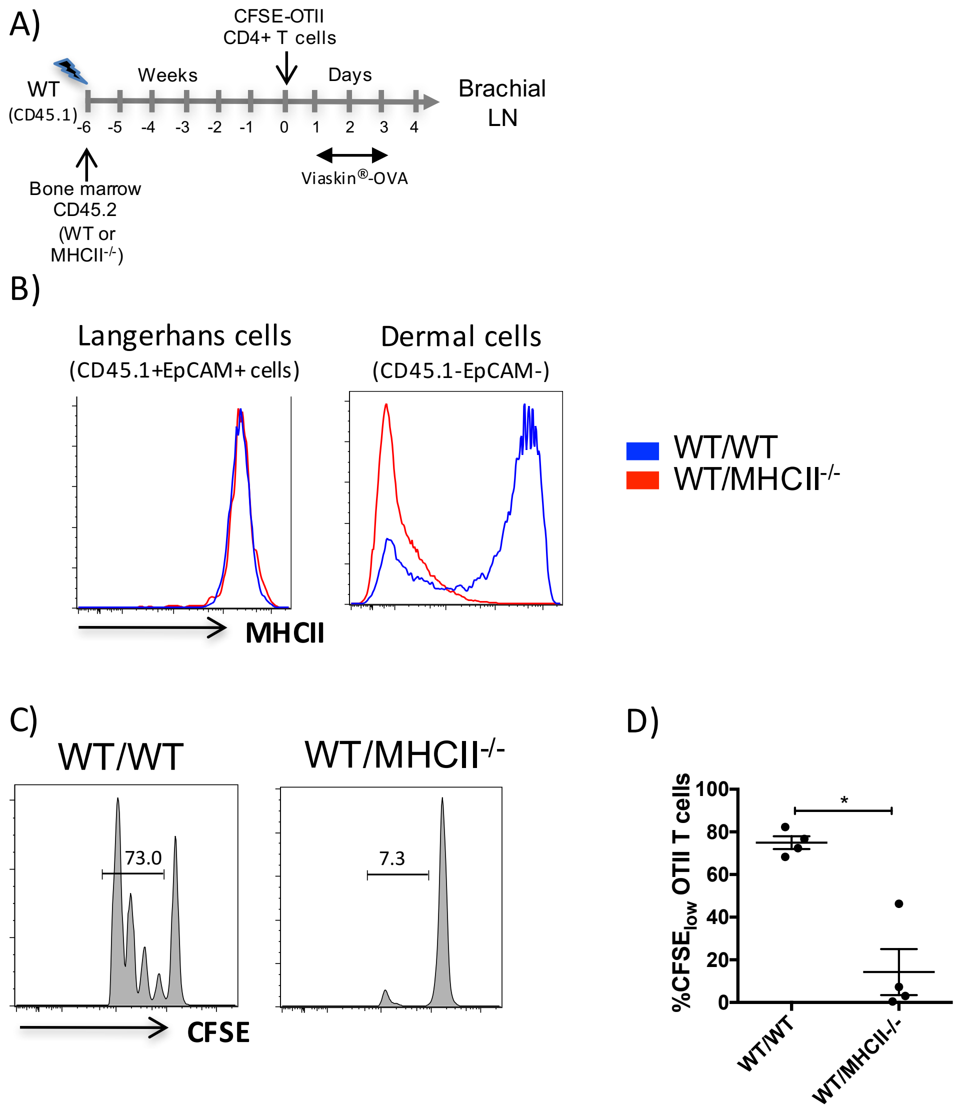

Supplementary Figure 4. Antigen-presentation capacity by bone marrow chimeras where all DCs or only Langerhans cells expressed MHCII. (A) Scheme of the experiment. (B) MHCII expression by LCs and dermal cells in the chimeras. (C) and (D) Percentage of $CFSE_{low}$ OT-II $CD4^{+}$ T cells recovered from brachial lymph nodes 3 days after application of OVA on bone marrow chimeras. Data are mean ± s.e.m. of 4 mice/group. *$p < 0.05$, Mann-Whitney test.

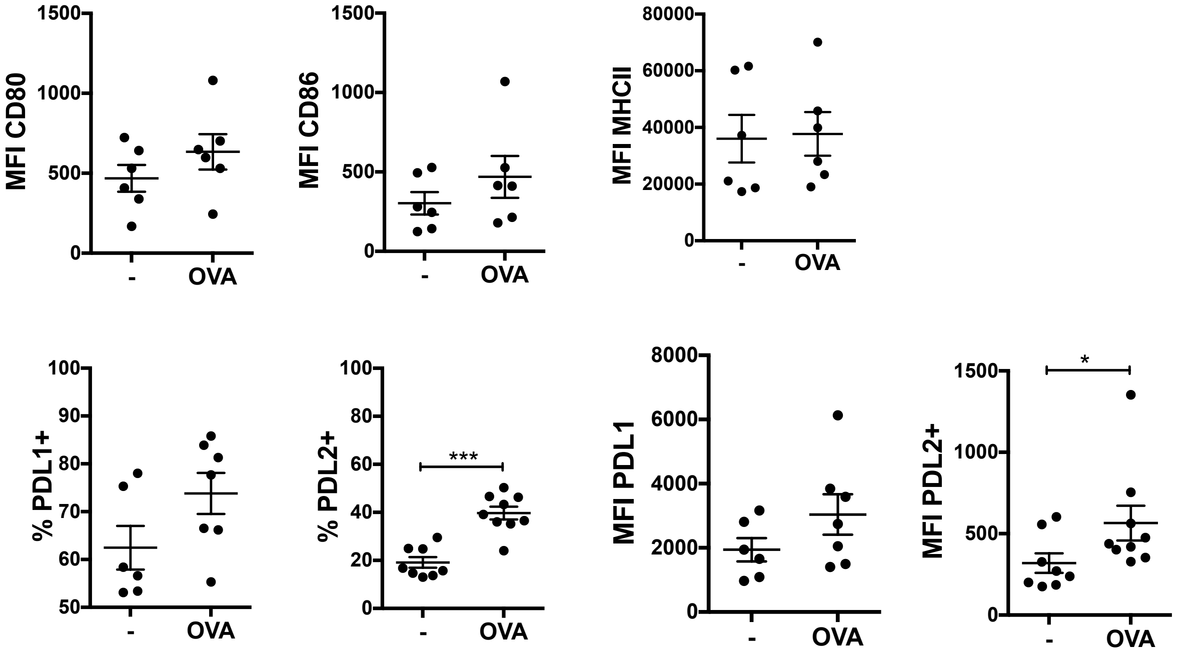

Supplementary Figure 5. Phenotype of migratory dendritic cells in mice untreated or mice treated with OVA patch. Median fluorescence intensity (MFI) or percentage (%) of $CD11c^{+}MHCII^{high}$ cells from skin-draining lymph nodes from Balb/c mice treated with OVA patch for 48h or untreated (-) mice. Data are mean ± s.e.m. of 6-9 mice/group. *p < 0.05, ***p < 0.001, Mann-Whitney test.

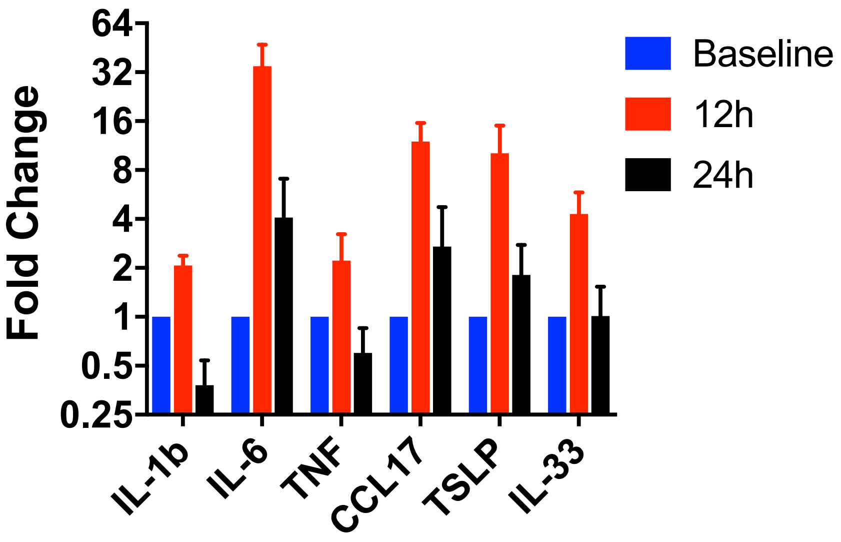

Supplementary Figure 6. Gene expression in skin after application of OVA patch. Skin was shaved and treated with depilatory 24 hours before application of a Viaskinpatch loaded with OVA. Dorsal skin was harvested at 12 or 24 hours and compared to skin from mice that did not receive the patch (baseline). N=3 mice per time point.

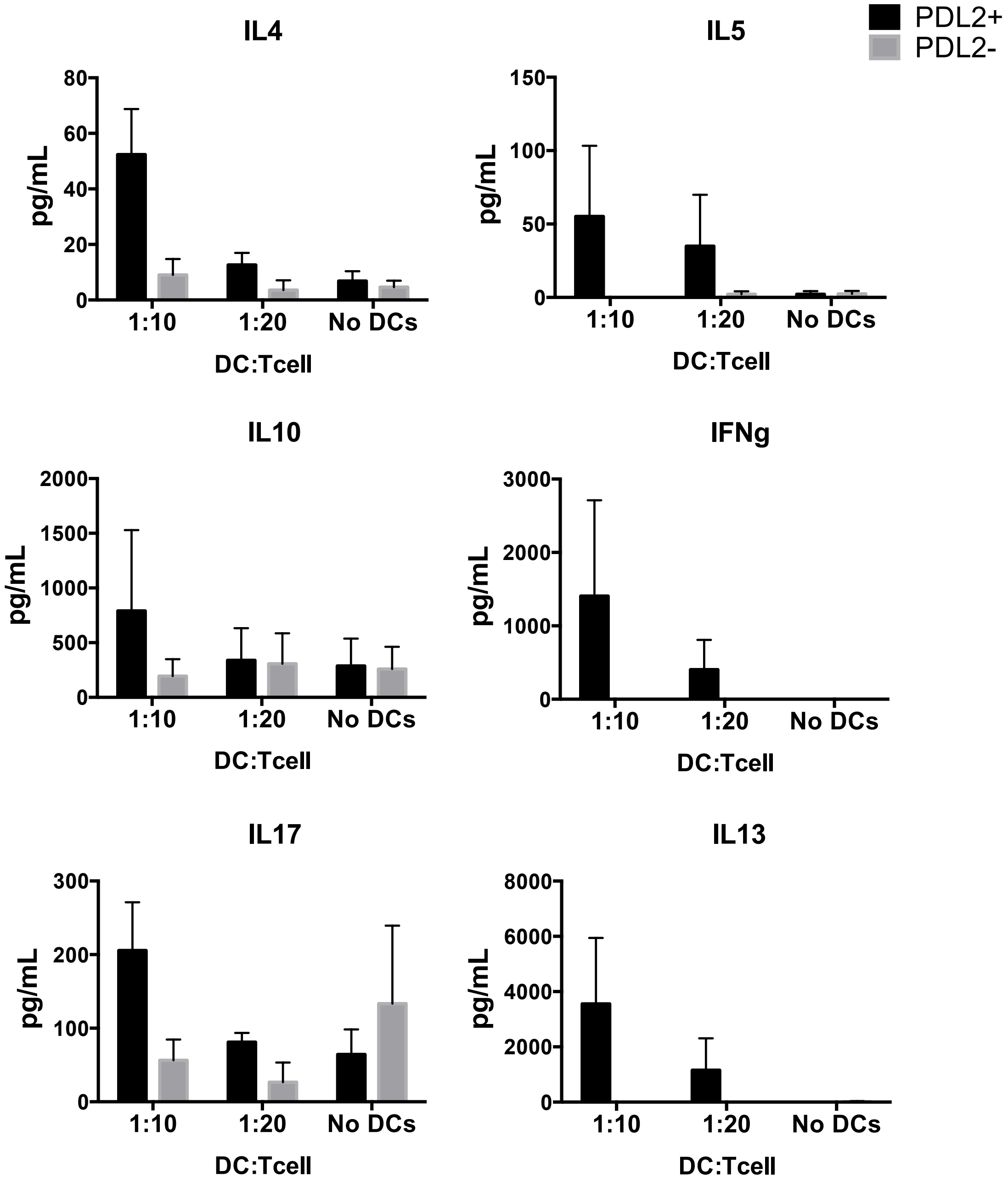

Supplementary Figure 7. PDL2+ DCs induce cytokine release by T cells after in vivo ***priming with OVA patch.*** PDL2+ (black) and PDL2- (grey) DCs were purified from the skin-draining lymph nodes of BALB/c mice exposed to OVA patch for 48h and co-cultured with DO11.10 CD4+ T-cells for 4 days, followed by re-stimulation with CD3/CD28. Representative graphics from 3 different experiments are shown.

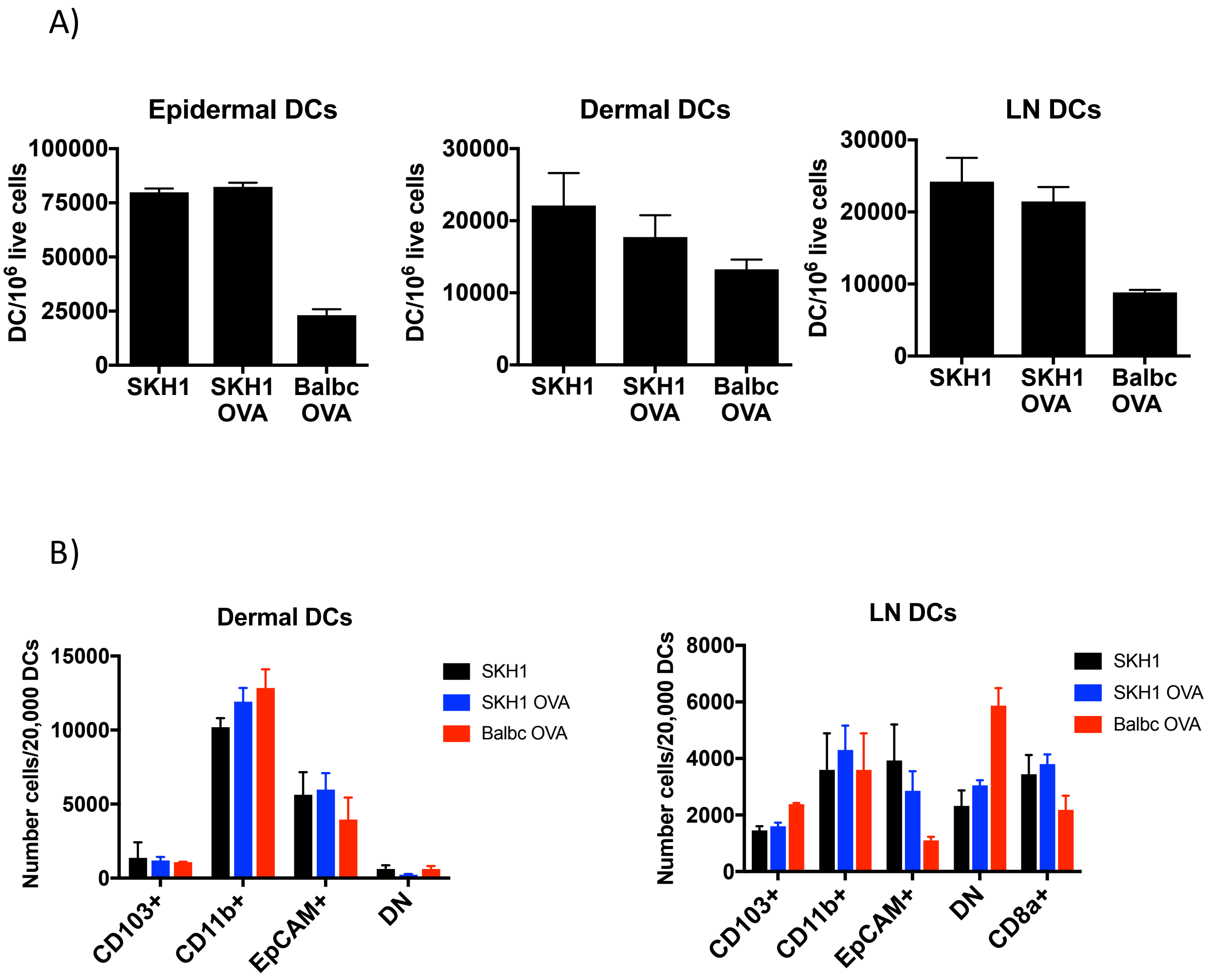

Supplementary Figure 8. Frequency of DCs in skin and lymph nodes of hairless SKH-1 mice and Balb/c mice. Cells were isolated from the epidermis, dermis, and brachial lymph nodes (LNs) of mice and stained for total DCs (A) and DC subsets (B).

**Condition**	**% CFSE low (range)**	**N**
No DCs	21.0 (4.6-38.4)	4
Total CD11c+	97.4 (90.3-99.1)	6
CD11b+	98.7 (94.5-99.9)	6
CD103+	96.8 (84.7-99.8)	6
EpCAM+	95.3 (77.7-99.9)	5
CD8+	19.7	1
Neg	55.2 (22.5-95.5)	3

Supplementary Table 1. Proliferation of DO11.10 T cells after co-culture with DC subsets loaded ex-vivo with OVA peptide 323-339.
