# Supplementary material for: PDL2+ CD11b+ dermal dendritic cells capture topical antigen through hair follicles to prime LAP+ Tregs
Source: Nat Commun. 2018 Dec 7;9:5238. doi: 10.1038/s41467-018-07716-7 (PMC6286332; doi:10.1038/s41467-018-07716-7)
Supplement: Supplementary file 3 — Description of Additional Supplementary Files [file 41467_2018_7716_MOESM3_ESM.pdf]

Description of Additional Supplementary Files

File Name: Supplementary Data 1
Description: Excel file providing data for Figure 1A,B,C

File Name: Supplementary Data 2
Description: Excel file providing data for Figure 2B,C,D,E,G

File Name: Supplementary Data 3
Description: Excel file providing data for Figure 3B

File Name: Supplementary Data 4
Description: Excel file providing data for Figure 4A-E

File Name: Supplementary Data 5
Description: Excel file providing data for Figure 5B-C

File Name: Supplementary Data 6
Description: Excel file providing data for Figure 6A-C

File Name: Supplementary Data 7
Description: Excel file providing data for Figure 7A,C

File Name: Supplementary Data 8
Description: Excel file providing data for Figure 8B,C
